# Outcomes of 434 dogs with non‐steroidal anti‐inflammatory drug toxicosis treated with fluid therapy, lipid emulsion, or therapeutic plasma exchange

**DOI:** 10.1111/jvim.16603

**Published:** 2022-12-01

**Authors:** Nolan V. Chalifoux, Emmanuelle M. Butty, Katie D. Mauro, Rachel B. Moyle, Caryn M. Ehrhardt, James B. Robertson, Mary A. Labato, Christine A. Culler, Leonel A. Londoño, Alessio Vigani, Yu Ueda, Steven E. Suter, Alex M. Lynch

**Affiliations:** ^1^ Department of Clinical Sciences & Advanced Medicine University of Pennsylvania, School of Veterinary Medicine Philadelphia Pennsylvania USA; ^2^ Department of Clinical Sciences Tufts University, Cummings School of Veterinary Medicine, Foster Hospital for Small Animals North Grafton Massachusetts USA; ^3^ Small Animal Clinical Sciences Michigan State University, College of Veterinary Medicine East Lansing Michigan USA; ^4^ BluePearl Pet Hospital Cary North Carolina USA; ^5^ Department of Small Animal Clinical Sciences University of Florida, College of Veterinary Medicine Gainesville Florida USA; ^6^ Department of Clinical Sciences North Carolina State University, College of Veterinary Medicine Raleigh North Carolina USA; ^7^ Capital Vet Specialists Jacksonville Florida USA; ^8^ Clinic for Small Animal Medicine, Vetsuisse Faculty University of Zurich Zurich Switzerland

**Keywords:** carprofen, ibuprofen, ILE, intoxication, naproxen, NSAID, TPE

## Abstract

**Background:**

Traditional management of non‐steroidal anti‐inflammatory drug (NSAID) intoxication includes gastrointestinal decontamination, intravenous administration of fluids (IVF), and gastroprotection. Intravenous administration of lipid emulsion (ILE) and therapeutic plasma exchange (TPE) are popular novel therapeutic strategies.

**Hypothesis:**

Compare outcomes of dogs treated with IVF, ILE, and TPE for NSAID intoxications and evaluate outcome predictors for drug subgroups.

**Animals:**

Four hundred thirty‐four dogs with NSAID intoxications (2015‐2020).

**Methods:**

Multicenter retrospective study of ibuprofen, carprofen, and naproxen intoxication. An ordinal outcome was defined as mild gastrointestinal, moderate kidney, or signs of severe central nervous system disease.

**Results:**

Signs of neurological disease were overrepresented and acute kidney injury underrepresented in the TPE group among dogs exposed to kidney‐ or CNS‐toxic doses (*P* = .05), though all TPE dogs with signs of neurological disease had evidence of neurotoxicity at presentation. Dogs treated with IVF had a higher maximal creatinine concentration (median, 1.1 mg/dL; range, 0.4‐8.44 mg/dL) compared with IVF + ILE (median, 0.9 mg/dL; range, 0.4‐6.2 mg/dL; *P* = .01). Increased maximum time to presentation (*P* < .001), higher baseline creatinine (*P* < .001) and PCV (*P* = .007), and absence of induced emesis (*P* < .001) were associated with greater clinical severity. Ibuprofen toxicosis was associated with more severe clinical signs compared with carprofen (*P* = .03). Overall survival rate was 99%.

**Conclusions and Clinical Importance:**

NSAID toxicosis generally carries an excellent prognosis in dogs. Despite similar outcomes of lower incidence of AKI in the TPE group, and slightly lower maximal creatinine concentration in dogs treated with ILE vs IVF alone, ILE and TPE should be considered in the management of severe NSAID toxicosis.

**Abbreviations**
AKI: acute kidney injury
CNS: central nervous system
COX: cyclooxygenase
GI: gastrointestinal
IVF: intravenous administration of fluids
ILE: intravenous administration of lipid emulsion
NSAID: non‐steroidal anti‐inflammatory drug
PG‐E1: prostaglandin E1
ROC: receiver operating characteristic
TPE: therapeutic plasma exchange

## 1 INTRODUCTION

Non‐steroidal anti‐inflammatory drugs (NSAIDs) are widely administered for managing acute and chronic pain conditions in human and veterinary medicine.^1^, ^2^, ^3^ Their high global prevalence has resulted in them being one of the most commonly reported poisonings in dogs,^1^, ^4^, ^5^, ^6^, ^7^ with dogs being the most commonly affected domesticated species.^1^ According to the American Society for the Prevention of Cruelty to Animals (ASPCA) Animal Poison Control Center (APCC), ibuprofen is the most frequently reported NSAID exposure in animals, followed by carprofen, aspirin, naproxen, meloxicam, and deracoxib.^1^ Clinical signs of acute NSAID intoxication range from mild gastrointestinal (GI) disturbances to severe kidney injury, with severe central nervous system (CNS) disturbances arising at maximal exposed doses.^1^, ^8^ Additionally, both dose‐dependent and idiosyncratic hepatoxicities occur.^1^, ^8^, ^9^ Traditional management of NSAID intoxications involves prompt gastrointestinal decontamination, administration of gastroprotectants, and the intravenous administration of fluids (IVF).^1^, ^8^

Over the past 10 years, intravenous administration of lipid emulsion (ILE) has gained popularity as a readily accessible and cost‐effective management strategy for several lipophilic drug intoxications in animals, including NSAIDs.^10^, ^11^, ^12^, ^13^ Therapeutic plasma exchange (TPE) has also become more commonly used in the management of NSAID intoxications in dogs.^14^, ^15^, ^16^, ^17^, ^18^, ^19^ This technique involves the separation of the cellular components of blood from plasma by either centrifugal or membrane‐based TPE, facilitated by either the rotation of a blood chamber or a nonselective membrane filter, respectively.^20^ This technique allows for high amounts of protein‐bound toxins to be removed from the circulation and replaced with donor plasma, preventing potentially life‐threatening consequences associated with NSAID intoxication.^18^, ^20^ To date, there are limited evidence‐based recommendations within the veterinary literature to guide clinicians in the management of NSAID intoxications with ILE and TPE.

The primary objective of this study was to compare outcomes of dogs treated with IVF therapy alone, or in combination with ILE, TPE, or ILE and TPE for the treatment of carprofen, ibuprofen, and naproxen intoxications. Secondary objectives were to evaluate predictors of outcome for specific drug subgroups and to investigate the sensitivity and specificity of potential thresholds among these predictors. We hypothesized that TPE would be associated with less severe clinical signs compared with IVF therapy alone, and that ILE would also be associated with positive outcomes, though to a lesser degree compared with TPE. Additionally, we hypothesized that time to presentation, maximal exposure dose, and naproxen ingestion would be associated with the development of more severe clinical signs.

## 2 MATERIALS AND METHODS

### 2.1 Case selection

Medical records of four veterinary teaching hospitals (College of Veterinary Medicine, University of Pennsylvania; the Cummings School of Veterinary Medicine, Tufts University; College of Veterinary Medicine, University of Florida; and College of Veterinary Medicine, North Carolina State University) and one private emergency and referral hospital (Blue Pearl Pet Hospital, North Carolina) were searched to identify dogs with NSAID ingestion treated with IVF alone, or in combination with ILE, TPE, or ILE and TPE between June 1, 2015 and June 30, 2020. Dogs of any age were considered for potential inclusion. Dogs that were discharged from the hospital and later re‐admitted for continued supportive care were excluded from the study for ease of data collection, in addition to dogs that were euthanized without treatment, or where elements of their medical records relevant to the study were incomplete. Dogs treated with other extracorporeal modalities (ie, hemoperfusion and hemodialysis) were excluded. Inclusion criteria were limited to ibuprofen, carprofen, and naproxen based on preliminary data review identifying the majority of NSAID intoxications involving these drugs.

### 2.2 Medical records review

Data recorded from the medical record included signalment, body weight, NSAID type, maximal NSAID dose, maximal time to presentation, clinical signs at presentation, and the following baseline and discharge clinical pathological variables: creatinine, blood urea nitrogen, phosphorus, sodium, potassium, alanine aminotransferase, aspartate aminotransferase, alkaline phosphatase, PCV, total solids, and urine specific gravity. Maximal NSAID dose was defined as the highest possible exposure dose reported in the history. Discharge clinical pathological data was defined as data last obtained before discharge from the hospital, or before death or humane euthanasia. Time to definitive treatment, induction of and success of emesis, and the following treatments were recorded: activated charcoal, H2‐receptor antagonist, proton pump inhibitor, and prostaglandin E1 (PG‐E1) analog. Predominant IVF rate, duration of IVF therapy, ILE bolus and CRI dose, if repeated ILE dosing was used, TPE platform, TPE plasma volumes exchanged, anticoagulation method, and TPE replacement solutions were recorded in addition to hospitalization time. Maximal creatinine concentration was recorded, defined as the highest creatinine concentration recorded by either the referring veterinarian or the referral hospital at any timepoint throughout hospitalization. To account for overlap among treatments, classification was adjusted to categorize dogs among one of four groups: IVF alone; IVF and ILE; IVF and TPE; or IVF, ILE, and TPE.

Dogs were classified according to their maximal exposure dose recorded within the medical record in mg/kg. For ibuprofen, GI, kidney, and CNS toxic doses were defined as >25 mg/kg, >100 mg/kg, and >400 mg/kg, respectively.^21^ For carprofen, GI, kidney, and CNS toxic doses were defined as >20 mg/kg, >40 mg/kg, and >200 mg/kg, respectively.^8^, ^21^ For naproxen, GI, kidney, and CNS toxic doses were defined as >5 mg/kg, >25 mg/kg, and >50 mg/kg, respectively.^21^

Signs of gastrointestinal disease were defined as clinical signs of vomiting, regurgitation, diarrhea, or dysrexia (anorexia or hyporexia). Acute kidney injury (AKI) was defined as a creatinine >1.6 mg/dL, or a rise in creatinine of ≥0.3 mg/dL during a 48‐hour time interval with a consistent history, clinical presentation and laboratory evaluation.^22^ Acute kidney injuries were graded according to The International Renal Interest Society AKI grading system for animals.^23^ Grade I non‐azotemic AKI was defined as creatinine ≤1.6 mg/dL with a rise in creatinine ≥0.3 mg/dL, grade II as creatinine 1.7 to 2.5 mg/dL, grade III as 2.6 to 5.0 mg/dL, grade IV as 5.1 to 10.0 mg/dL, and grade V as >10.0 mg/dL. Baseline creatinine values were obtained either from the referring veterinarian, or the referral hospital as available. Signs of neurological disease were defined as the presence of seizure activity, ataxia, or objective evidence of intracranial disease such as abnormal cranial nerve function or an altered state of consciousness.

Survival to discharge was defined as discharge with no recorded rehospitalization for persistent clinical signs pertaining to the same intoxication event. One‐year survival was recorded and defined as cases where confirmatory record of euthanasia, death, or survival was present among the medical record 1‐year after the date of discharge.

### 2.3 Statistical methods

Statistical analyses were conducted using R version 4.0.3 with packages *AER*, *ggplot2*, and *MASS*. The Shapiro‐Wilk test was used to assess continuous variables for normality. Descriptive statistics consisted of the median and range for all continuous variables given that the majority of the variables were not normally distributed. The count and percentage (%) were used to report frequency data.

For the purpose of examining associations between severity of signs and recorded variables, a cumulative logistic regression model was fit with no clinical signs as the best, followed by signs of GI disease, AKI, and then signs of neurological disease as the worst. Cumulative logistic regression models are used to predict an ordinal response under the assumption of proportional odds, with parallel slopes across all levels of the response. No adjustment was made for animals with multiple clinical signs, they were judged solely by their worst clinical sign for the purpose of statistical modeling. Multivariate models were constructed with forward selection with an entry criteria of *P* < .10 in an ANOVA comparing the current model to the model with an additional term. The term with the lowest *P*‐value that was not also related to an existent term was selected while only considering terms that would not reduce the number of complete cases drastically (at least a 30% reduction in cases, a complete case being one which has values for all parameters in the model). The multivariate logistic regression models were formulated using variables that were considered to be potentially significant at the univariate level with a coefficient *P*‐value <0.10 as the criteria (Table S1). Time to presentation, time to treatment, maximal exposure dose, baseline creatinine, maximal creatinine concentration, and baseline BUN were transformed using log_10_. Additional models investigating the association between treatment group and outcome were generated after case stratification by GI, kidney, and CNS maximal exposure toxic doses.

Continuous variables were compared between groups using the Kruskal‐Wallis test and Fisher's exact test was used to compare proportions. Bonferroni's correction was applied to account for pairwise multiple comparisons of treatment groups. The area under the receiver operating characteristic (ROC) curve was calculated as a nonparametric estimation with DeLong confidence intervals. Confidence intervals for sensitivity and specificity were done via bootstrapping with 2000 resamples. A cut‐off value was then selected to maximize the sensitivity and specificity of significant variable thresholds with the goal of establishing a clinically relevant cut‐off for likely development of clinical signs. For all comparisons, *P* < .05 was considered statistically significant.

## 3 RESULTS

### 3.1 Animals

A total of 434 dogs (199 Tufts University, 101 Blue Pearl Pet Hospital, 53 University of Pennsylvania, 42 University of Florida, 39 North Carolina State University) were included in the study. The median age and body weight of all dogs on admission to the hospital was 3 years (range, 2 months to 16 years) and 18 kg (range, 1‐60 kg), respectively. One hundred ninety one (44%) dogs were castrated males, 171 (39%) dogs were spayed females, 37 (9%) dogs were intact females, and 34 (8%) dogs were intact males. One dog was classified as a female of unknown spay status. One hundred thirteen (26%) dogs were classified as mixed breed dogs and 321 (74%) dogs were classified as purebreds. Notable breeds included 54 (12%) Labrador retrievers, 22 (5%) dachshunds, 17 (4%) beagles, 15 (3%) golden retrievers, 14 (3%) German shepherds, 11 (3%) boxers, 11 (3%) bulldogs, 10 (2%) Chihuahuas, and 9 (2%) Australian shepherds.

Two hundred twenty‐five (52%) dogs were presented for ingestion of ibuprofen, 129 (30%) dogs were presented for ingestion of carprofen, and 80 (18%) dogs were presented for ingestion of naproxen. Historical, clinical, and clinicopathological findings and treatment summaries are summarized in Tables 1, 2, 3 respectively. The median time from NSAID ingestion to maximal treatment level was 5 hours (range, 0.2‐168 hours) for IVF alone and 4 hours for all IVF + ILE (range, 0.3‐48 hours), IVF + TPE (range, 1‐48 hours), and IVF + ILE + TPE (range, 1‐22 hours) treatment groups (*P* = .17).

**TABLE 1:** Historical and clinical signs at presentation of dogs presented for ibuprofen, carprofen, and naproxen toxicosis

	Ibuprofen		Carprofen		Naproxen		Total	
	Proportion (%)	Median (range)	Proportion (%)	Median (range)	Proportion (%)	Median (range)	Proportion (%)	Median (range)
Dogs	225/434 (52)		129/434 (30)		80/434 (18)		434/434 (100)	
Gastrointestinal toxic dose	212/212 (100)		121/125 (97)		76/76 (100)		409/413 (99)	
Renal toxic dose	164/212 (77)		99/125 (79)		63/76 (83)		326/413 (79)	
Central nervous toxic dose	70/212 (33)		15/125 (12)		46/76 (61)		131/413 (32)	
Signs of gastrointestinal disease	104/225 (46)		28/129 (22)		22/80 (28)		154/434 (35)	
Acute kidney injury	41/225 (18)		7/129 (5)		5/80 (6)		53/434 (12)	
Signs of neurological disease	12/225 (5)		0/129 (0)		2/80 (3)		14/434 (3)	
Maximal exposure dose (mg/kg)		206 (25, 4857)		78 (11, 625)		82 (9, 3000)		
Maximal time to presentation (hours)		5 (0.3, 168)		4 (0.5, 125)		3.6 (0.3, 144)		4 (0.3, 168)

*Note*: Acute kidney injury (AKI) was defined as a creatinine >1.6 mg/dL, or a rise in creatinine of ≥0.3 mg/dL during a 48‐hour time interval, with a consistent history, clinical presentation and laboratory evaluation.

**TABLE 2:** Clinicopathological findings at presentation of dogs presented for ibuprofen, carprofen, and naproxen toxicosis

	Ibuprofen	Carprofen	Naproxen	Total
	Median (range)	Median (range)	Median (range)	Median (range)
Creatinine (mg/dL)	1.1 (0.2, 6.9)	1 (0.5, 6.4)	0.9 (0.4, 4.4)	1.0 (0.2, 7.0)
Blood urea nitrogen (mg/dL)	18 (7, 213)	19 (4, 196)	20 (8, 93)	19 (4, 213)
Phosphorus (mg/dL)	5.3 (2.9, 16)	4.6 (2.2, 17.8)	4.5 (2.0, 9.8)	4.9, (2.0, 17.8)
Sodium (mmol/L)	147 (122, 160)	148 (125, 161)	147 (139, 160)	147 (122, 161)
Potassium (mmol/L)	4.3 (3.1, 5.9)	4.2 (1.7, 5.5)	4.3 (3.2, 5)	4.3 (1.7, 5.9)
ALT (U/L)	63 (11, 502)	68 (10, 1637)	54 (27, 470)	64 (10, 1637)
AST (U/L)	46 (18, 166)	44 (20, 755)	43 (29, 450)	45 (18, 755)
ALP (U/L)	63 (<10, 688)	60 (<10, 2062)	69 (<10, 993)	62 (<10, 2062)
PCV (%)	50 (33, 66)	48 (24, 62)	48 (27, 66)	50 (24, 66)
Total solids (g/dL)	6.8 (4.2, 9.5)	6.7 (5.0, 9.3)	6.6 (3.5, 10.2)	6.7 (3.5, 10.2)
Urine specific gravity	1.026 (1.006, 1.060)	1.041 (1.006, 1.060)	1.050 (1.008, 1.060)	1.034 (1.006, 1.060)

Abbreviations: ALP, alkaline phosphatase; ALT, alanine aminotransferase; AST, aspartate aminotransferase.

**TABLE 3:** Treatment summary of dogs presented for ibuprofen, carprofen, and naproxen toxicosis

	Ibuprofen	Carprofen	Naproxen	Total
	Proportion (%)	Proportion (%)	Proportion (%)	Proportion (%)
Induced emesis	146/223 (65)	101/129 (78)	54/80 (68)	301/432 (70)
Successful emesis	141/144 (98)	92/97 (95)	50/53 (94)	283/294 (96)
Activated charcoal	145/224 (65)	97/129 (75)	61/80 (76)	303/433 (70)
H2 antagonist	52/225 (23)	15/128 (12)	21/80 (27)	88/433 (20)
Proton pump inhibitor	213/225 (95)	118/129 (91)	76/80 (95)	407/434 (94)
PG‐E_1_ analog	136/224 (61)	81/129 (63)	50/80 (63)	267/433 (62)
IVF alone	164/225 (73)	100/129 (78)	46/80 (58)	310/434 (71)
IVF + ILE	33/225 (15)	15/129 (12)	15/80 (19)	63/434 (15)
IVF + TPE	15/225 (7)	13/129 (10)	10/80 (13)	38/434 (9)
IVF + ILE + TPE	13/225 (6)	1/129 (1)	9/80 (11)	23/434 (5)

Abbreviations: ILE, intravenous administration of lipid emulsion; IVF, intravenous administration of fluids; PG, prostaglandin; TPE, therapeutic plasma exchange.

### 3.2 Fluid therapy and lipid emulsion

The median IVF rate for all dogs was 5 mL/kg/hr (range, 1‐10 mL/kg/hr). For dogs that underwent ILE therapy, the median ILE bolus dose was 1.5 mL/kg (range, 1.5‐3 mL/kg) followed by a median continuous rate infusion (CRI) of 0.25 mL/kg/min (range, 0.16‐6.1 mL/kg/min) for all dogs. The median CRI duration was 1.5 hours (range, 0.75‐3 hours). Five (6%) dogs received a second ILE bolus and CRI throughout hospitalization. The median total ILE dose was 16.5 mL/kg (range, 1.5‐542 mL/kg).

### 3.3 Therapeutic plasma exchange

For dogs that underwent TPE, 36 (59%) treatments were performed on a membrane‐based platform and 25 (41%) were performed on a centrifuge‐based platform. Dogs undergoing membrane‐based TPE received systemic anticoagulation with unfractionated heparin, while dogs undergoing centrifuge‐based TPE received regional citrate anticoagulation in all cases in addition to unfractionated heparin in 19 of 25 (76%) dogs. The median plasma volumes exchanged was 1.6 (range, 0.4‐2.2) over a median treatment duration of 2 hours (range, 1‐4.5 hours). Fifty‐nine (97%) dogs received donor plasma as part of their replacement solution, 34 (56%) dogs received isotonic crystalloids, and 32 (52%) dogs received synthetic colloids. Two (3%) dogs had their TPE treatment discontinued before completion resulting in <1 plasma volumes exchanged. One dog had a transmembrane pressure elevation of unknown cause and a subsequent filter leak after replacement of the TPE set. The second dog had also had a transmembrane pressure elevation of unknown cause and severe hypotension. One dog received two TPE treatments.

### 3.4 Clinical signs

Data pertaining to GI, kidney, and CNS dysfunction throughout hospitalization for ibuprofen, carprofen, and naproxen toxicosis are summarized in Table 4, and the presence of clinical signs throughout hospitalization for each treatment group are summarized in Table 5. Dogs treated with IVF alone developed signs of GI disease, AKI, and signs of neurological disease that were not present at presentation in 72 of 310 (23%), 31 of 310 (10%), and 1 of 310 (0.3%) cases, respectively. Dogs treated with IVF and ILE developed signs of GI disease, AKI, and signs of neurological disease that were not present at presentation in 14 of 63 (22%), 5 of 63 (8%), and 1 of 63 (2%) cases, respectively. Dogs treated with IVF + TPE developed signs of GI disease, AKI, and signs of neurological disease that were not present at presentation in 12 of 38 (32%), 9 of 38 (24%), and 0 of 38 (0%) cases, respectively. Dogs treated with IVF + ILE + TPE developed signs of GI disease, AKI, and signs of neurological disease that were not present at presentation in 8 of 23 (35%), 6 of 23 (26%), and 0 of 23 (0%) cases, respectively.

**TABLE 4:** Summary of gastrointestinal, renal, and central nervous system dysfunction noted at any point throughout hospitalization of dogs presented for ibuprofen, carprofen, and naproxen toxicosis

	Ibuprofen		Carprofen		Naproxen		Total	
	Proportion (%)	Median (range)	Proportion (%)	Median (range)	Proportion (%)	Median (range)	Proportion (%)	Median (range)
Signs of gastrointestinal disease	131/219 (60)		57/129 (44)		32/79 (41)		220/427 (52)	
Acute kidney injury	75/217 (35)		15/127 (12)		10/79 (13)		100/423 (24)	
Maximal creatinine (mg/dL)		1.2 (0.4, 8.4)		1 (0.4, 6.4)		0.9 (0.4, 4.4)		1.1 (0.4, 8.44)
IRIS AKI								
Grade I	16/75 (21)		4/15 (27)		4/10 (40)		24/100 (24)	
Grade II	29/75 (39)		5/15 (33)		4/10 (40)		38/100 (38)	
Grade III	26/75 (35)		5/15 (33)		2/10 (20)		33/100 (33)	
Grade IV	4 /75 (5)		1/15 (7)		0/10 (0)		5/100 (5)	
Grade V	0/75 (0)		0/15 (0)		0/10 (0)		0/100 (0)	
Signs of neurological disease	12/219 (5)		1/129 (1)		0/78 (0)		13/426 (3)	

*Note*: Acute kidney injury (AKI) was defined as a creatinine >1.6 mg/dL, or a rise in creatinine of ≥0.3 mg/dL during a 48‐hour time interval, with a consistent history, clinical presentation, and laboratory evaluation. Grade I non‐azotemic AKI was defined as creatinine ≤1.6 mg/dL with a rise in creatinine ≥0.3 mg/dL, grade II as creatinine 1.7 to 2.5 mg/dL, grade III as 2.6 to 5.0 mg/dL, grade IV as 5.1 to 10.0 mg/dL, and grade V as >10.0 mg/dL.

**TABLE 5:** Summary of gastrointestinal, renal, and central nervous system dysfunction noted at any point throughout hospitalization of dogs presented for ibuprofen, carprofen, and naproxen toxicosis treated with a combination of intravenous administration of fluids, lipid emulsion, or therapeutic plasma exchange

	IVF alone	IVF + ILE	IVF + TPE	IVF + ILE + TPE
	Proportion (%)	Proportion (%)	Proportion (%)	Proportion (%)
Signs of gastrointestinal disease	163/305 (53)	27/61 (44)	17/38 (45)	13/23 (57)
Acute kidney injury	73/305 (24)	9/61 (15)	11/38 (29)	7/23 (30)
Signs of neurological disease	4/305 (1)	4/61 (7)	2/38 (5)	3/23 (13)

Abbreviations: ILE, intravenous administration of lipid emulsion; IVF, intravenous administration of fluids; TPE, therapeutic plasma exchange.

### 3.5 Univariate analysis: Treatment intervention

A significant association was found between treatment and most extreme clinical signs for all dogs, where more severe clinical signs throughout hospitalization were associated with ILE and TPE provision (*P* = .03). However, a significant association was also found between treatment group and level of toxic dose (ie, GI vs kidney vs CNS), where a higher exposed dose was associated with an escalation in treatment complexity (*P* < .001). Dogs treated with IVF alone had a significantly higher maximal creatinine concentration (median, 1.1 mg/dL; range, 0.4‐8.44 mg/dL) at any point throughout hospitalization compared with dogs treated with IVF + ILE (median, 0.9 mg/dL; range, 0.4‐6.2 mg/dL; *P* = .01). No significant difference in hospitalization length (*P* = .06) or survival to discharge (*P* = .15) was found between individual treatment groups. However, dogs undergoing TPE had significantly longer hospitalization times (median, 54 hours; range, 24‐264 hours) compared with dogs that did not undergo TPE (median, 48 hours; range, 0‐290 hours; *P* = .04).

### 3.6 Univariate exposure dose stratification analysis: treatment intervention

Among dogs that consumed any NSAID at a renal‐toxic dose, no significant association between TPE usage and severity of clinical signs was identified (*P =* .42). Among dogs that consumed any NSAID at a CNS‐toxic dose, no significant association between TPE usage and severity of clinical signs was identified (*P =* .55). Among dogs that consumed any NSAID at a renal‐ or CNS‐toxic dose, a significant association between TPE usage and severity of clinical signs was identified with signs of CNS disease overrepresented and acute kidney injury underrepresented in the TPE group (*P* = .05). This remained true after subgroup analysis for ibuprofen (*P* = .02), but not for carprofen (*P* = .71) and naproxen (*P* = .84). All dogs with signs of neurological disease that underwent TPE already had evidence of neurotoxicity at presentation. One dog had resolution of signs of neurological disease during TPE and all others ultimately recovered from their signs of neurological disease after treatment.

Among dogs that consumed any NSAID at a renal‐toxic dose, no significant association between IVF + ILE usage and severity of clinical signs was identified compared with IVF alone (*P* = .46). For dogs that consumed any NSAID at a CNS‐toxic dose, no significant association between IVF + ILE usage and severity of clinical signs was identified compared with IVF alone (*P* = .84). Among dogs that consumed any NSAID at a renal‐ or CNS‐toxic dose, no significant association between IVF + ILE usage and severity of clinical signs was identified compared with IVF alone (*P* = .31). Among dogs that consumed a renal‐ or CNS‐toxic dose of any NSAID and underwent TPE, the additional use of ILE was not associated with the severity of clinical signs (*P* = .84).

### 3.7 Multivariate analysis: Predictors of outcome and clinical thresholds

**FIGURE 1:**
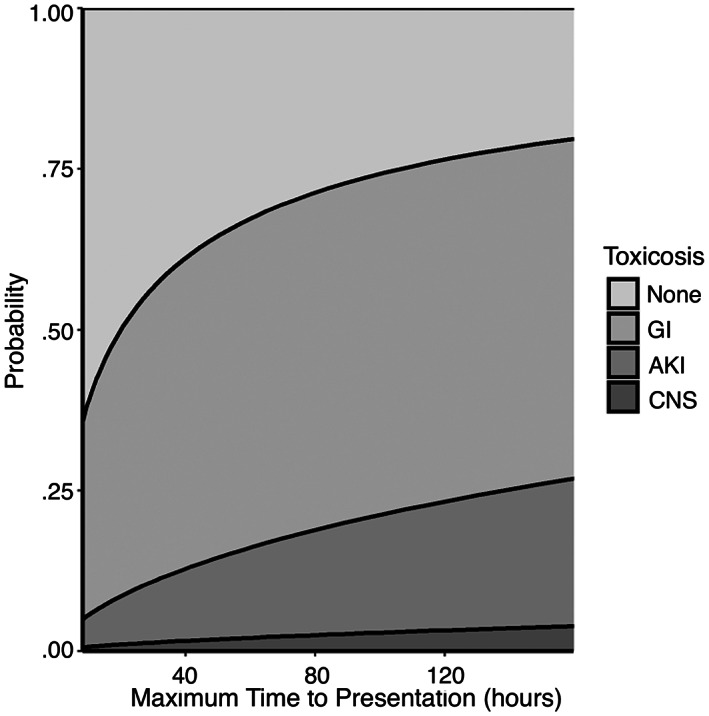
Stacked area chart displaying the effect of maximal time to presentation (hours) on the cumulative probability of developing signs of gastrointestinal disease, acute kidney injury, and neurological disease in dogs presented for non‐steroidal anti‐inflammatory toxicosis after cumulative logistic regression modeling. AKI, acute kidney injury; CNS, central nervous system; GI, gastrointestinal

**FIGURE 2:**
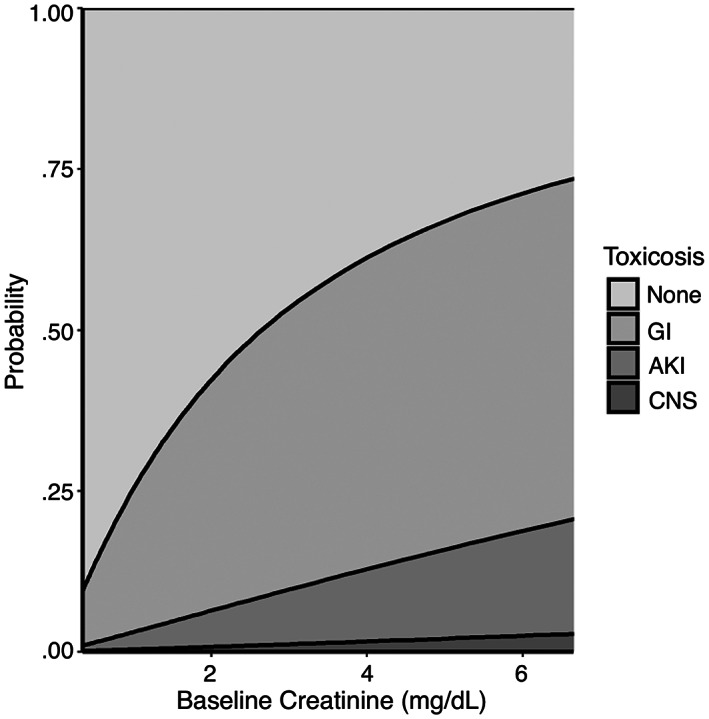
Stacked area chart displaying the effect of baseline creatinine (mg/dL) on the cumulative probability of developing signs of gastrointestinal, acute kidney injury, and neurological disease in dogs presented for non‐steroidal anti‐inflammatory toxicosis after cumulative logistic regression modeling. AKI, acute kidney injury; CNS, central nervous system; GI, gastrointestinal

**FIGURE 3:**
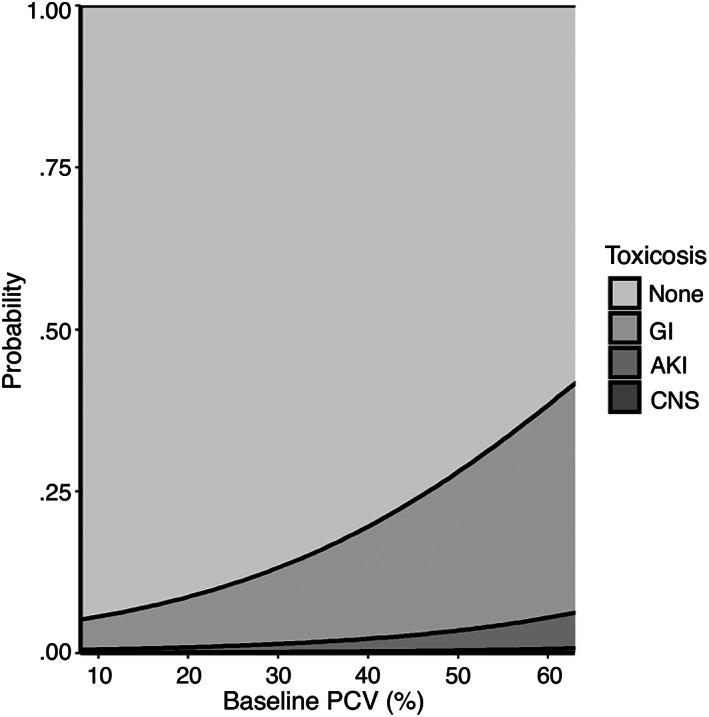
Stacked area chart displaying the effect of baseline packed cell volume (%) on the cumulative probability of developing signs of gastrointestinal, acute kidney injury, and neurological disease in dogs presented for non‐steroidal anti‐inflammatory toxicosis after cumulative logistic regression modeling. AKI, acute kidney injury; CNS, central nervous system; GI, gastrointestinal

**FIGURE 4:**
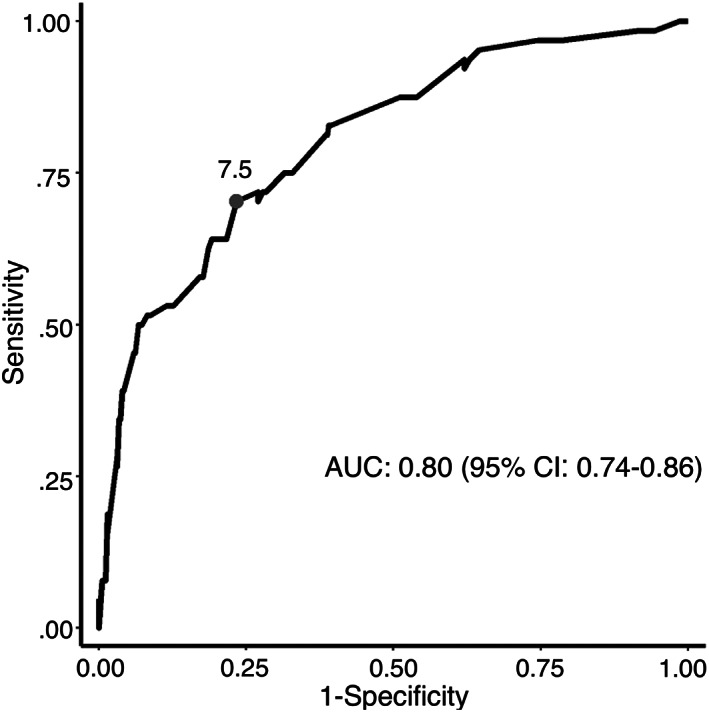
Receiver operating characteristic (ROC) curve for maximal time to presentation to predict the development of an acute kidney injury in dogs presented for non‐steroidal anti‐inflammatory toxicosis. AUC, area under the curve; the identified point represents a maximal time to presentation threshold of 7.5 hours that was found to have a maximized sensitivity and specificity for the prediction of an acute kidney injury or signs of central nervous system disease. Dogs diagnosed with an acute kidney injury at presentation were not excluded from the ROC analysis

**FIGURE 5:**
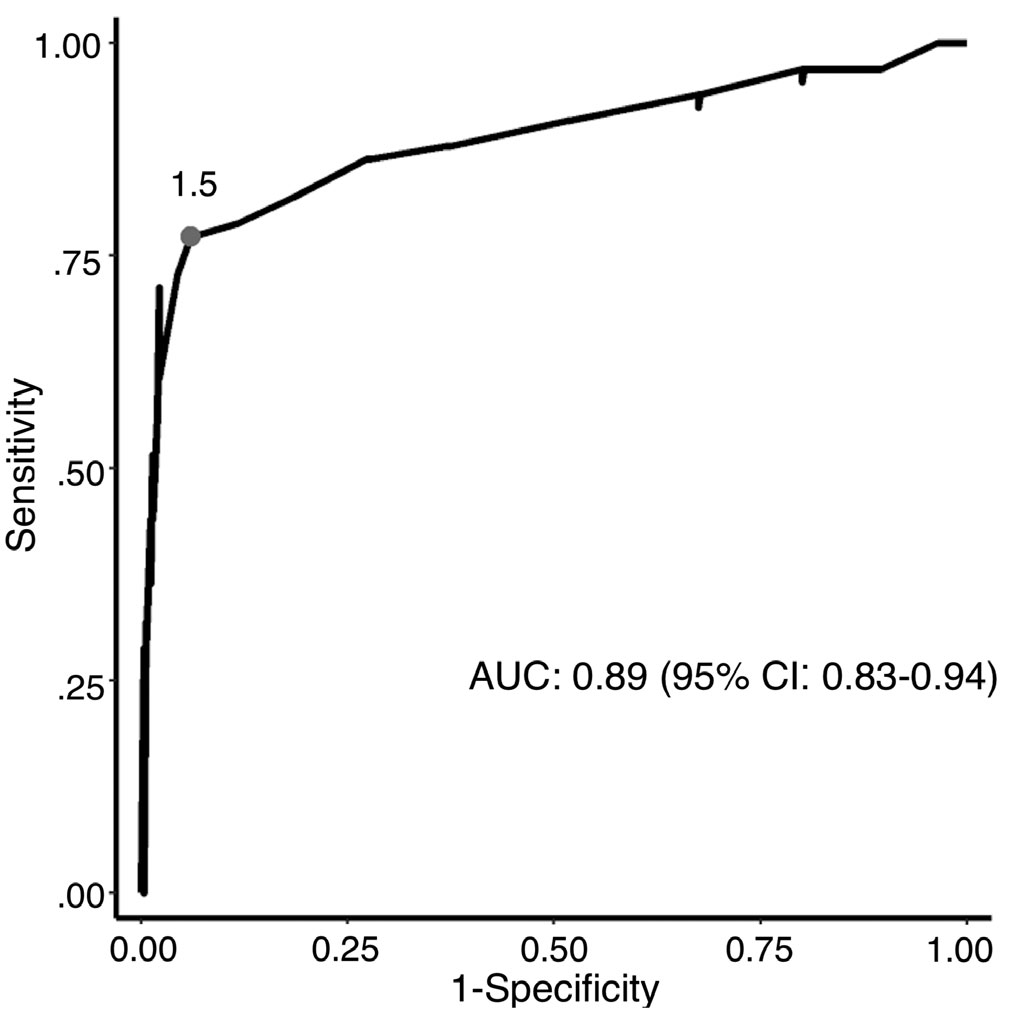
Receiver operating characteristic (ROC) curve for baseline creatinine concentration at presentation to predict the development of an acute kidney injury in dogs presented for non‐steroidal anti‐inflammatory toxicosis. AUC, area under the curve; the identified point represents a baseline creatinine threshold of 1.5 mg/dL that was found to have a maximized sensitivity and specificity for the prediction of an acute kidney injury or signs of central nervous system disease. Dogs diagnosed with an acute kidney injury at presentation were not excluded from the ROC analysis

**FIGURE 6:**
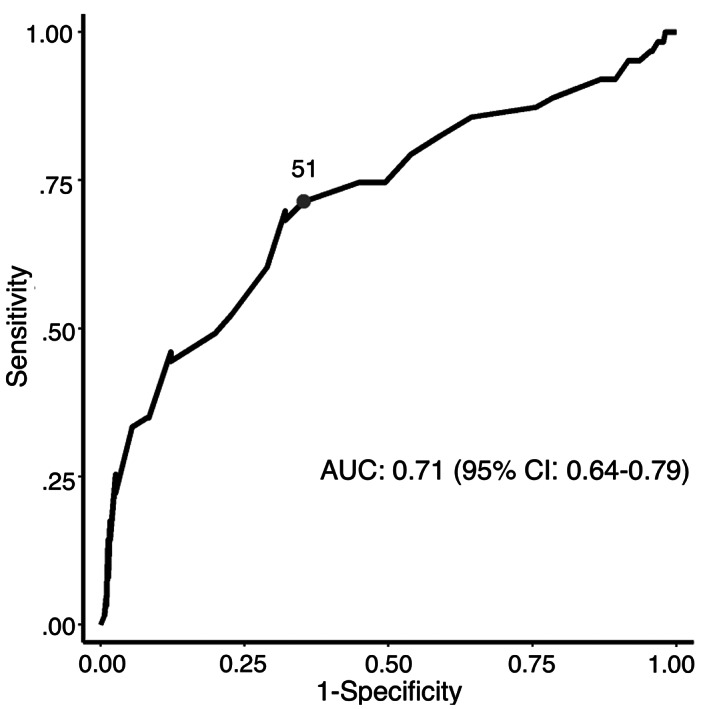
Receiver operating characteristic (ROC) curve for packed cell volume percentage at presentation to predict the development of an acute kidney injury in dogs presented for non‐steroidal anti‐inflammatory toxicosis. AUC, area under the curve; the identified point represents a packed cell volume threshold of 51% that was found to have a maximized sensitivity and specificity for the prediction of an acute kidney injury or signs of central nervous system disease. Dogs diagnosed with an acute kidney injury at presentation were not excluded from the ROC analysis

**FIGURE 7:**
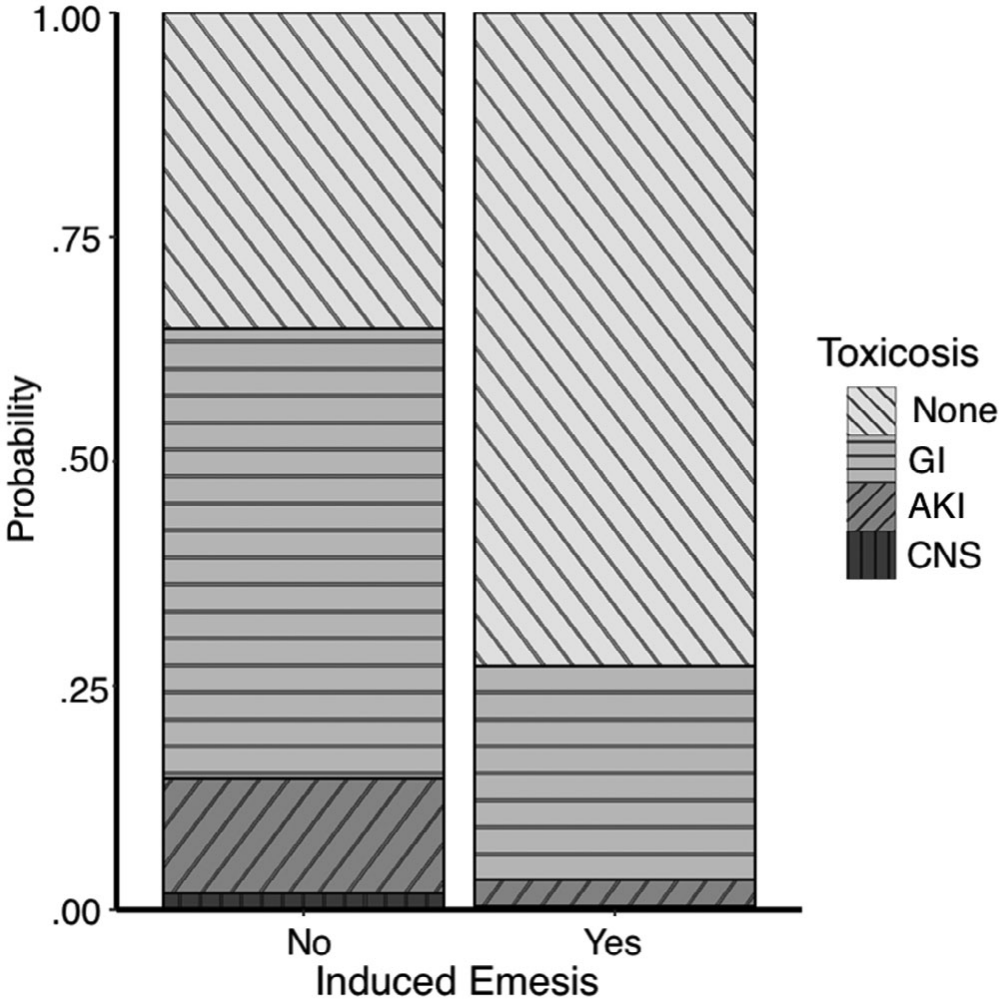
Stacked bar chart displaying the association of emesis induction on the cumulative probability of developing signs of gastrointestinal, acute kidney injury, and neurological disease in dogs presented for non‐steroidal anti‐inflammatory toxicosis after cumulative logistic regression modeling. AKI, acute kidney injury; CNS, central nervous system; GI, gastrointestinal

An increase in the following variables was found to be associated with the development of more severe clinical signs: maximum time to presentation (Figure 1, *P* < .001), baseline creatinine (Figure 2, *P* < .001), and baseline PCV (Figure 3, *P* = .007). A threshold of 7.5 hours as a maximal time to presentation predicted the development of AKI with a maximized sensitivity of 70% (95% CI: 55%‐80%) and a specificity of 77% (95% CI: 61%‐84%; Figure 4). The area under the ROC curve was 80% (95% CI: 74%‐86%). A baseline creatinine threshold of 1.5 mg/dL predicted the development of AKI with a maximized sensitivity of 77% (95% CI: 65%‐86%) and a specificity of 94% (95% CI: 72%‐99%; Figure 5). The area under the ROC curve was 89% (95% CI: 83%‐94%). A baseline PCV threshold of 51% predicted the development of AKI with a maximized sensitivity of 70% (95% CI: 52%‐80%) and a specificity of 68% (95% CI: 43%‐75%; Figure 6). The area under the ROC curve was 71% (95% CI: 64%‐79%). Induction of emesis was significantly associated with better clinical signs (Figure 7, *P* < .001).

### 3.8 Ibuprofen, carprofen, and naproxen subgroup analysis

**FIGURE 8:**
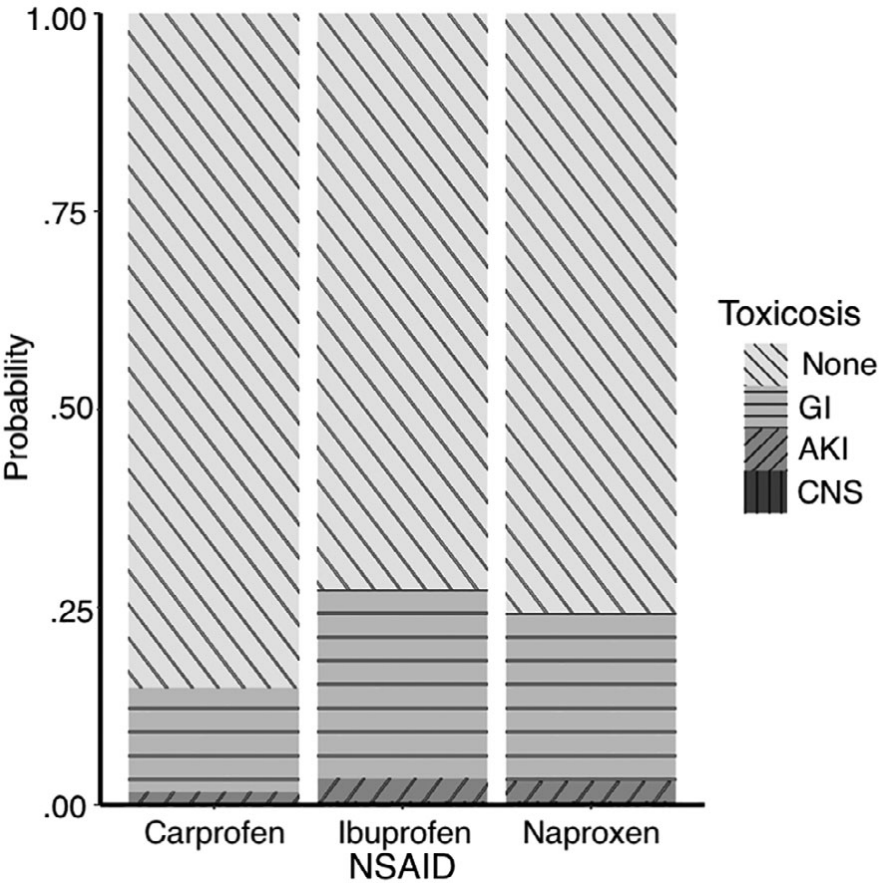
Stacked bar chart displaying the association of non‐steroidal anti‐inflammatory drug (NSAID) on the cumulative probability of developing signs of gastrointestinal, acute kidney injury, and neurological disease in dogs presented for NSAID toxicosis after cumulative logistic regression modeling. AKI, acute kidney injury; CNS, central nervous system; GI, gastrointestinal

**FIGURE 9:**
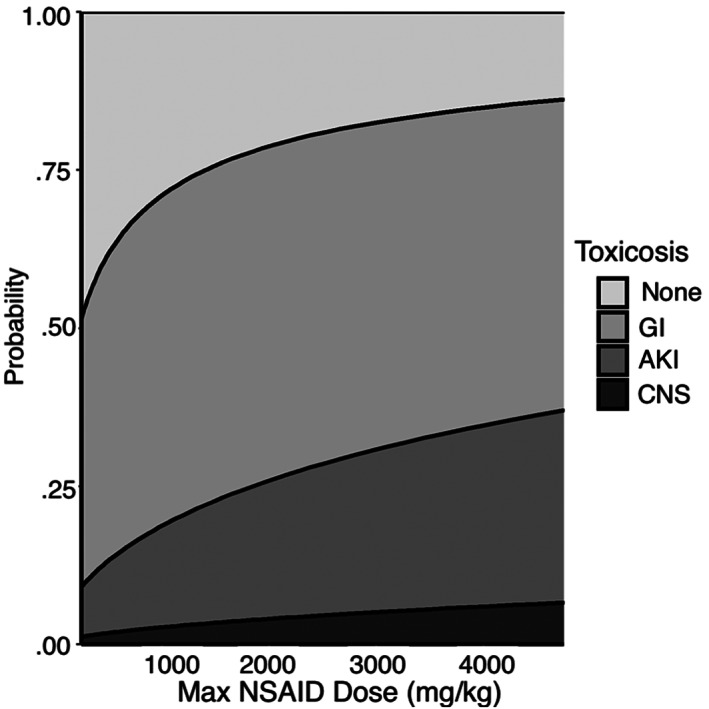
Stacked area chart displaying the effect of maximal exposure dose (mg/kg) on the cumulative probability of developing signs of gastrointestinal, acute kidney injury, and neurological disease in dogs presented for ibuprofen toxicosis after cumulative logistic regression modeling. AKI, acute kidney injury; CNS, central nervous system; GI, gastrointestinal

Ibuprofen toxicosis was significantly associated with the development of more severe clinical signs compared with the ingestion of carprofen (Figure 8, *P* = .03). Among subgroup analysis for ibuprofen toxicosis, maximal exposure dose was found to be associated with the development of more severe clinical signs (Figure 9, *P* < .001). A maximal exposure dose threshold of 400 mg/kg predicted the development of AKI with a maximized sensitivity of 47% (95% CI: 22%‐60%) and a specificity of 71% (95% CI: 46%‐78%). The area under the ROC curve was 56% (95% CI: 47%‐66%). This association was not identified for carprofen and naproxen. Additionally, dogs treated with IVF + ILE were associated with the development of more severe clinical signs compared with those treated with IVF alone (*P* = .01), though significance was lost after exposure dose stratification (*P* = .39).

Among subgroup analysis for naproxen toxicosis, a decrease in body weight was found to be significantly associated with more severe clinical signs (*P* = .009), while higher maximal creatinine concentrations were significantly associated with better clinical signs (*P* = .004). A body weight threshold of ≤7.1 kg predicted the development of AKI with a maximized sensitivity of 57% (95% CI: 14%‐86%) and a specificity of 81% (95% CI: 32%‐97%). The area under the ROC curve was 56% (95% CI: 43%‐91%). The incidence of AKI was 8% (95% CI: 3%‐18%) and 28% (95% CI: 10%‐53%) in dogs above (N = 61) and below (N = 18) 7.1 kg, respectively. No additional significant associations were identified among subgroup analysis for carprofen toxicosis.

### 3.9 Survival and follow‐up

Total survival rate was 99% with 429 out of 434 total dogs surviving to discharge. Three non‐survivors received IVF therapy alone, 1 dog received IVF therapy and ILE, and 1 dog received IVF therapy and TPE. All non‐survivors ingested carprofen with the exception of 1 dog treated with IVF therapy and ILE that ingested ibuprofen. Among 79 dogs with follow‐up data available at 1‐year after discharge, 69 (87%) dogs were documented to still be alive.

## 4 DISCUSSION

The aim of this multicenter retrospective study was to compare the outcome of dogs treated with IVF therapy alone, or in combination with ILE, TPE, or ILE and TPE for the treatment of NSAID intoxications. Dogs exposed to high kidney‐ or CNS‐toxic doses of NSAIDs had a lower incidence of AKI throughout hospitalization when treated with TPE, and the addition of ILE to fluid therapy was associated with a lower maximal creatinine concentration. Though signs of neurological disease were overrepresented among dogs exposed to kidney‐ or CNS‐toxic doses of NSAIDs and treated with TPE, this reflected the degree of toxicosis rather than intervention as all dogs were presented with neurological derangements before therapy. Dogs receiving ILE or TPE had more severe clinical signs throughout hospitalization when maximal exposure dose was not considered. However, this finding was no longer significant after stratification for exposure dose.

Fifteen percent of dogs treated with IVF + ILE in our study developed kidney injury, while 7% developed signs of neurological disease. The use of ILE was found to reduce the maximal creatinine concentration in dogs compared with those treated with IVF alone. However, the clinical relevance of this finding is questionable as both medians remained within the normal range for dogs. In vitro lipemia has a negative bias on creatinine measurement in dogs, which might have contributed to this finding.^24^, ^25^ However, post‐prandial lipemia does not markedly influence serum creatinine concentrations in dogs.^26^

Kidney injury was present in ~30% of dogs treated with TPE in our study. The incidence of AKI was lower among this cohort if exposed to kidney‐ or CNS‐toxic doses compared with dogs that did not undergo TPE. Signs of neurological disease were present in 5% and 13% of dogs treated with IVF + TPE and IVF + ILE + TPE, respectively. All dogs with signs of neurological disease that were treated with TPE had signs of neurological disease at presentation. Dogs treated with TPE had significantly longer hospitalization times in our study, but the median hospitalization time was only 6 hours longer which is unlikely to be clinically important. The median hospitalization time for dogs undergoing TPE was 2.25 days, similar to a mean of 2.8 days for dogs undergoing TPE for NSAID overdose.^18^

Increased maximum time to presentation was associated with the development of more severe clinical signs, with a threshold >7.5 hours being a moderate predictor for AKI development. A similar association exists between a longer time from ingestion to intervention with a greater risk of GI ulceration and severe AKI in dogs treated conservatively for ibuprofen toxicosis.^27^ While a recent study failed to identify an association between outcome and maximum time to presentation after multivariate analysis, increasing time from ingestion to admission was associated with the development of both AKI and suspected GI ulceration at the univariate level.^28^

Packed cell volume has been associated with the duration of hospitalization in dogs undergoing TPE for NSAID overdoses, where dogs with lower PCV had longer hospitalization times.^18^ Low PCV might indicate hemorrhage secondary to GI ulceration or be associated with circuit loss as a complication of TPE.^18^ Contrarily, our study found that a higher PCV was associated with the development of more severe clinical signs throughout hospitalization, with a PCV ≥51% at presentation serving as a moderate to weak predictor of AKI. This association might reflect hemoconcentration, where dehydration might have occurred secondary to GI losses and reflect a greater disease severity at the time of presentation.

Induction of emesis was associated with the development of fewer clinical signs throughout hospitalization. Previously, induction of emesis before TPE therapy for NSAID toxicosis in dogs did not significantly change outcome.^18^ Charcoal administration in hospital is associated with a lower odds of AKI development at the univariate level, though this association did not persist at the multivariate level.^28^ Although these results might have been biased against emesis induction in dogs with more severe signs of neurological disease, they do support its use as a component of decontamination in cases of NSAID intoxication when appropriate.

Ibuprofen has greater potential for morbidity in dogs compared with other NSAIDs,^1^, ^12^, ^16^, ^21^, ^29^, ^30^ while the nonselective nature of naproxen and its long half‐life of 74 hours has been highlighted throughout the literature as a cause for concern.^1^, ^14^, ^16^, ^31^ Our study found that ibuprofen toxicosis was associated with the development of more severe clinical signs compared carprofen but not naproxen. Similarly, others have not identified a significant difference between ibuprofen and naproxen toxicosis with regards to the development of AKI or signs of GI disease in dogs undergoing TPE.^18^

While a threshold of 400 mg/kg was found to have a maximized sensitivity and specificity for the development of AKI among dogs with ibuprofen toxicosis, it was assessed to have a poor predictive value. Other studies have failed to find an association between maximal exposure dose and the development of AKI or signs of GI disease in dogs undergoing TPE.^18^ However, NSAID dose was found to be associated with maximal creatinine concentration during hospitalization and a larger change in creatinine concentration from baseline.^18^ A dose‐response relationship has also not been identified for acute ibuprofen toxicosis in dogs treated with traditional therapy with relation to GI ulceration and severe AKI.^27^

While a dose‐response relationship was not identified for cases of carprofen and naproxen toxicosis in our study, this might represent a type II error secondary to lower case numbers. Alternatively, subjective concerns regarding naproxen toxicosis might have contributed to TPE being utilized in approximately twice as many naproxen cases as compared with ibuprofen, which might have hindered the ability to identify an association with maximal exposure dose for naproxen. Additionally, this might have prevented the development of more severe clinical signs for naproxen ingestion compared with other NSAIDs, as was initially hypothesized.

Decreased body weight was associated with more severe clinical signs; however, this was only true among subgroup analysis for naproxen toxicosis. A possible explanation for this association might include a greater predisposition to dehydration in smaller dogs,^32^ mirroring the possible association between hemoconcentration at presentation and more severe outcome. A body weight threshold of ≤7.1 kg had a moderate specificity of 81% for the development of AKI after naproxen intoxication. However, a low sensitivity of 57% was associated with this marker suggesting a poor clinical applicability of this threshold. No associations between different body weights in dogs with acute ibuprofen toxicosis and subsequent development of organ dysfunctions have been described.^27^ However, broad size group categories in this study and relatively low power might have introduced bias and potential type II error.^27^ Similarly, no association between weight and survival, AKI, or GI ulceration was identified in a more recent retrospective evaluation of 125 dogs with NSAID overdose.^28^

While the findings of this multicenter study serve to aid in the management of NSAID toxicosis in veterinary medicine, it is not without limitations. Importantly, none of the cases included in this study had serum drug concentrations measured and none of the NSAID intoxications were confirmed. As the ordinal outcome applied in this study was inherently confounded by a dog's status at presentation, the modeling approach used was limited by its inability to account for clinical signs at presentation that persisted throughout hospitalization. Subsequently, this resulted in artifacts when interpreting the odds of clinical sign development for individual animals. Furthermore, it is also important to recognize the limitations associated with the high number of statistical comparisons performed in this study, despite the application of a Bonferroni correction to pairwise comparisons of treatment groups. As such, the authors caution overinterpretation of this study's significant findings as they might be associated with a type I statistical error. All but one veterinary hospital was equipped to perform TPE. As such, it might not be appropriate to extrapolate findings among this cohort of dogs to all veterinary facilities. Given the cost associated with increasing treatment intervention, an unconscious selection bias might also be present in this cohort of dogs related to owner financial capabilities. Moreover, this selection bias is complicated by the phenomenon of confounding‐by‐indication, where dogs exposed to higher toxic doses were more likely to be treated with either ILE or TPE, because of a greater clinical concern for morbidity and mortality.^33^ As a retrospective study, the data acquired are inherently limited by their availability and accuracy within the medical record. Time to presentation and maximal exposure dose were provided as estimates and were associated with a margin of error. Comorbidities and a comprehensive list of medications were also not evaluated. Baseline data pertaining to evidence of AKI at presentation was limited by an inability to adequately distinguish pre‐renal and renal azotemia in many cases. Furthermore, creatinine and BUN are insensitive markers of kidney function. As such, early kidney injury might not be detected, and improvement or decline in kidney function is hard to assess. The accuracy of clinical pathological data was also limited by the use of multiple analytical devices. Assessment of GI dysfunction was limited to clinical signs in this study and the presence of subclinical GI changes cannot be ruled out. With regards to outcome analysis, the high survival rate of 99% means there was insufficient power to evaluate for associations with death, and lack of true drug exposure might have contributed to this finding. This is similar to the previous reported survival rate of 96% for NSAID overdose in dogs.^28^

Non‐steroidal anti‐inflammatory drug toxicosis generally carries an excellent prognosis in dogs and despite similar overall outcomes, ILE and TPE should be considered in the management of severe NSAID toxicosis.

## CONFLICT OF INTEREST DECLARATION

Authors declare no conflict of interest.

## OFF‐LABEL ANTIMICROBIAL DECLARATION

Authors declare no off‐label use of antimicrobials.

## INSTITUTIONAL ANIMAL CARE AND USE COMMITTEE (IACUC) OR OTHER APPROVAL DECLARATION

Authors declare no IACUC or other approval was needed.

## HUMAN ETHICS APPROVAL DECLARATION

Authors declare human ethics approval was not needed for this study.

## Supporting information


**Table S1.** Variables considered for inclusion in cumulative logistic regression models and their significance.
