# Supplementary figures and images for: Outcomes of 434 dogs with non‐steroidal anti‐inflammatory drug toxicosis treated with fluid therapy, lipid emulsion, or therapeutic plasma exchange
Source: J Vet Intern Med. 2022 Dec 1;37(1):161–72. doi: 10.1111/jvim.16603 (PMC9889694; doi:10.1111/jvim.16603)

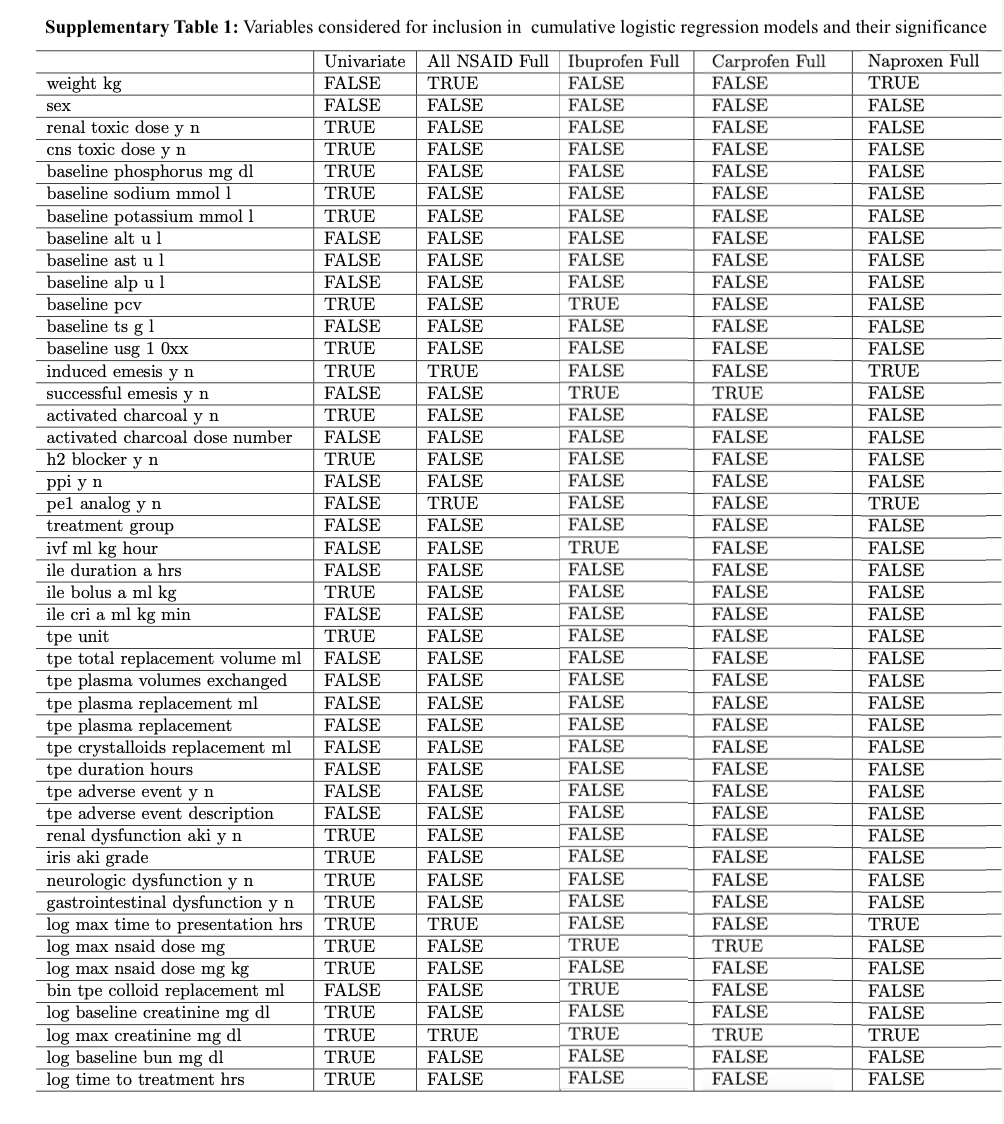

Supplement: Supplementary file 1 — Table S1. Variables considered for inclusion in cumulative logistic regression models and their significance. [file JVIM-37-161-s001.png]
